# New concepts of chitosan-based mucosal formulations for supporting control of the gastric pathogen *Helicobacter pylori*

**DOI:** 10.3389/fmicb.2026.1937380

**Published:** 2026-09-09

**Authors:** Patrycja Jaroniek, Maria Połomska, Magdalena Chmiela, Weronika Gonciarz

**Affiliations:** 1 Department of Immunology and Infectious Biology, Institute of Microbiology, Biotechnology and Immunology, Faculty of Biology and Environmental Protection, University of Lodz, Lodz, Poland; 2 BioMedChem Doctoral School of the University of Lodz and Lodz Institutes of the Polish Academy of Sciences, University of Lodz, Lodz, Poland

**Keywords:** antibiotic resistance, chitosan carriers, gastric environment, gastric tissue regeneration, *Helicobacter pylori*, immune reprogramming, *M. bovis* BCG, mucosal immunity

## Abstract

*Helicobacter pylori* (*H. pylori*) is a human gastric pathogen that colonizes the stomach by adapting to acidic pH, penetrating the gastric mucus layer, adhering to epithelial and mucosal receptors, and delivering virulence factors that promote inflammation and immune evasion. Antibiotic-based regimens have markedly reduced the burden of *H. pylori*-associated disease; however, increasing antimicrobial resistance, treatment failure, recurrence, and disruption of the gut microbiota remain important clinical challenges. Future therapeutic strategies should therefore combine bacterial control with modulation of the gastric environment that sustains chronic infection and impaired host responses. Chitosan-based micro- and nanoparticles are promising delivery systems for drugs and biologically active compounds because of their biodegradability, cationic surface charge, and mucoadhesive properties. This Mini Review summarizes chitosan-based formulations proposed for the delivery of antibiotics, plant-derived antimicrobial compounds, and antimicrobial peptides, as well as systems targeting *H. pylori* adhesins, bacterial biofilm, or urease activity. We also discuss an experimental concept in which the immunomodulatory vaccine strain *Mycobacterium bovis* Bacillus Calmette-Guerin (BCG) is encapsulated in chitosan micro- or nanoparticles for gastric and/or intestinal delivery. Available *in vitro* and *in vivo* evidence suggests that chitosan-based systems may increase local exposure to active components, support mucosal immune responses, and potentially facilitate epithelial repair. Nevertheless, most data remain preclinical, and clinical efficacy in humans has not been demonstrated. The review highlights the need for broader independent validation, careful biosafety assessment, and standardized formulation parameters before these approaches can be considered for translation.

## 1 Introduction

*Helicobacter pylori* is a Gram-negative, spiral-shaped, microaerophilic bacterium adapted to the gastric niche through urease activity, motility, and the ability to persist within the mucus layer. *H. pylori* can modulate the activity of selected immune cells and affect the gastric microbiota (Chmiela et al., 1997; Paziak-Domańska et al., 2000; Grebowska et al., 2008; Rudnicka et al., 2015; Gonciarz et al., 2023b; Malfertheiner et al., 2022; Ansari and Yamaoka, 2019). Persistent *H. pylori* infection is associated with chronic gastritis, peptic ulcer disease, and non-cardia gastric cancer (Malfertheiner et al., 2022; IARC Working Group, 1994; Plummer et al., 2015).

The management of *H. pylori* infection is increasingly challenging because of resistance to clarithromycin, metronidazole, and levofloxacin, which reduces treatment success. Current therapeutic options in regions with high antibiotic resistance include 14-day bismuth quadruple therapy based on a proton pump inhibitor, bismuth, tetracycline, and metronidazole, whereas non-bismuth quadruple therapy (proton pump inhibitor, amoxicillin, clarithromycin, and metronidazole) is reserved for selected cases. Clarithromycin-based triple therapy is no longer recommended empirically and should be used only when susceptibility to this antibiotic has been confirmed. In selected patients, bismuth quadruple therapy, rifabutin-based regimens, or potassium-competitive acid blockers such as vonoprazan-amoxicillin may be advantageous (Malfertheiner et al., 2022; Yu et al., 2024; Schulz et al., 2025; Chey et al., 2024).

Treatment failure can result from antimicrobial resistance, poor patient adherence, or insufficient acid suppression (Malfertheiner et al., 2022; Schulz et al., 2025). Antibiotic therapy may also disturb the gut microbiota and impair immune balance and gastric tissue recovery (Huang et al., 2024; Albush et al., 2025). These limitations support the search for strategies that reduce antibiotic pressure, preserve the microbiota, and modulate host immune responses. Formulations capable of delivering biologically active components to the stomach, where *H. pylori* persists, are therefore of particular interest. Chitosan-based nanoparticles (NPs) and microparticles (MPs) are attractive carriers for gastrointestinal delivery because of their mucoadhesive properties and ability to encapsulate antimicrobial, immunomodulatory, or pro-regenerative cargo (Cheung et al., 2015). In this context, chitosan particles loaded with the immunomodulatory vaccine strain *Mycobacterium bovis* Bacillus Calmette-Guerin (BCG) represent a relatively unexplored but promising preclinical approach. In the *Cavia porcellus* model, orally administered chitosan carriers loaded with *M. bovis* BCG facilitated cargo release in the stomach and gut and activated immunocompetent cells involved in the control of *H. pylori* colonization (Gonciarz et al., 2024a, 2026a). Thus, chitosan should not be viewed only as a passive carrier, because its mucoadhesive, antimicrobial, and immunomodulatory properties may also support anti-*H. pylori* strategies.

## 2 Gastric environment supporting *H. pylori* persistence

*H. pylori* has developed several mechanisms that enable colonization of the gastric mucosa despite the acidic and mucus-rich environment of the stomach. The bacterium can migrate using flagella, locally neutralize gastric acidity through urease activity, and adhere to mucin 5AC (MUC5AC) or other mucus and epithelial receptors (Backert et al., 2011; Chmiela and Kupcinskas, 2019; Baj et al., 2021; Clyne et al., 2025). Adhesion involves several bacterial factors, including blood group antigen-binding adhesin (BabA), sialic acid-binding adhesin (SabA), Helicobacter outer membrane protein Q (HopQ), outer inflammatory protein A (OipA), and other outer membrane proteins, whose expression may change in response to local gastric conditions (Ilver et al., 1998; Mahdavi et al., 2002; Bäckström et al., 2004; Aspholm et al., 2006; Baj et al., 2021; Backert et al., 2011).

*H. pylori* can form biofilms that limit antibiotic penetration, protect bacteria from environmental stress, and promote the transition of some bacteria into metabolically less active states (Elshenawi et al., 2023). Therefore, antibiotic susceptibility assessed in planktonic bacteria may not fully reflect the sensitivity of the total bacterial population. Key virulence factors associated with epithelial damage and inflammation include cytotoxin-associated gene A protein (CagA), which is delivered into host cells via the type IV secretion system and interferes with cell signaling, polarization, proliferation, and the hummingbird phenotype (Segal et al., 1999), and vacuolating toxin A (VacA), which affects mitochondrial function, promotes autophagy or apoptosis, and interferes with immunocompetent cell activity (Baj et al., 2021; Caron et al., 2015). Together with lipopolysaccharide, mucolytic and proteolytic enzymes, and factors released or delivered through outer membrane vesicles, these virulence determinants sustain chronic inflammation, oxidative stress, epithelial damage, abnormal proliferation, apoptosis, and delayed regeneration (Chmiela et al., 2014; Caron et al., 2015; Gonciarz et al., 2019). Therefore, therapeutic intervention should target not only *H. pylori* itself but also the restoration of mucosal homeostasis.

## 3 Concepts of chitosan-based mucosal therapeutic formulations

Chitosan is one of the most widely studied biopolymers for designing therapeutic formulations for mucosal delivery because it combines biodegradability, biocompatibility, chemical modifiability, and the ability to form MPs, NPs, hydrogels, or polyelectrolyte complexes (Gonciarz et al., 2025; Aranaz et al., 2021; Mikušová and Mikuš, 2021). In the context of *H. pylori* infection, chitosan mucoadhesion and its positive surface charge, resulting from protonated amino groups, enable interactions with negatively charged mucosal components, bacterial surfaces, and epithelial cell membranes (Gonciarz et al., 2025; Mikušová and Mikuš, 2021; Aibani et al., 2021). In practice, chitosan formulations can prolong the contact of active substances with the gastric mucosa, which is important because rapid gastric emptying, acidic pH, and mucus turnover can limit the effectiveness of orally administered drugs. Chitosan can protect active components from degradation, increase their stability, and enable controlled local release (Gonciarz et al., 2025; Aranaz et al., 2021; Mikušová and Mikuš, 2021). It can therefore be used to load antibiotics, natural biologically active compounds, or antimicrobial peptides. Depending on molecular weight, degree of deacetylation, solubility, and surface charge, chitosan may also influence bacterial viability on mucosal and epithelial surfaces. Studies on *H. pylori* have shown that chitosan itself can inhibit bacterial growth, urease activity, and adhesion to gastric epithelium (Chang et al., 2020).

Moreover, chitosan can modulate mucosal immune responses through adjuvant-like properties (Gonciarz et al., 2025). This was shown in studies using chitosan-derived carriers loaded with *M. bovis* BCG, in which chitosan enhanced retention, protected the payload in acidic conditions, and improved antibacterial or immune effects of mycobacteria after per os administration to *Cavia porcellus* (Gonciarz et al., 2024a). In this context, chitosan is an interesting biomaterial that combines passive delivery functions with potential biological activity.

Several chemically modified chitosan derivatives may also be useful for designing anti-*H. pylori* formulations. These include carboxymethyl chitosan/poly-gamma-glutamic acid sponges that facilitate prolonged gastric contact, thiolated chitosan that enhances mucoadhesion through disulfide interactions with mucus glycoproteins, and trimethylated chitosan that increases cationic charge and strengthens interactions with mucus, bacterial membranes, and epithelial surfaces (Zhang et al., 2025). However, the choice of native or modified chitosan should be adjusted to the intended release site, cargo type, and safety requirements, because derivatization can affect degradation, toxicity, batch-to-batch reproducibility, and regulatory classification.

Overall, chitosan-based NPs and MPs intended for oral application offer several advantages, including biocompatibility, biodegradability, mucoadhesion, cargo protection, and controlled local release in the stomach, where acidic pH, mucus turnover, and rapid clearance can reduce drug activity. However, these formulations also have limitations related to molecular weight, degree of deacetylation, particle size, zeta potential, pH-dependent solubility, and manufacturing method. Highly cationic formulations may improve mucosal interactions, but they may also increase cytotoxicity or excessive immune activation. Scale-up, sterilization, long-term stability, and reproducibility remain important barriers to clinical translation.

**Figure 1:**
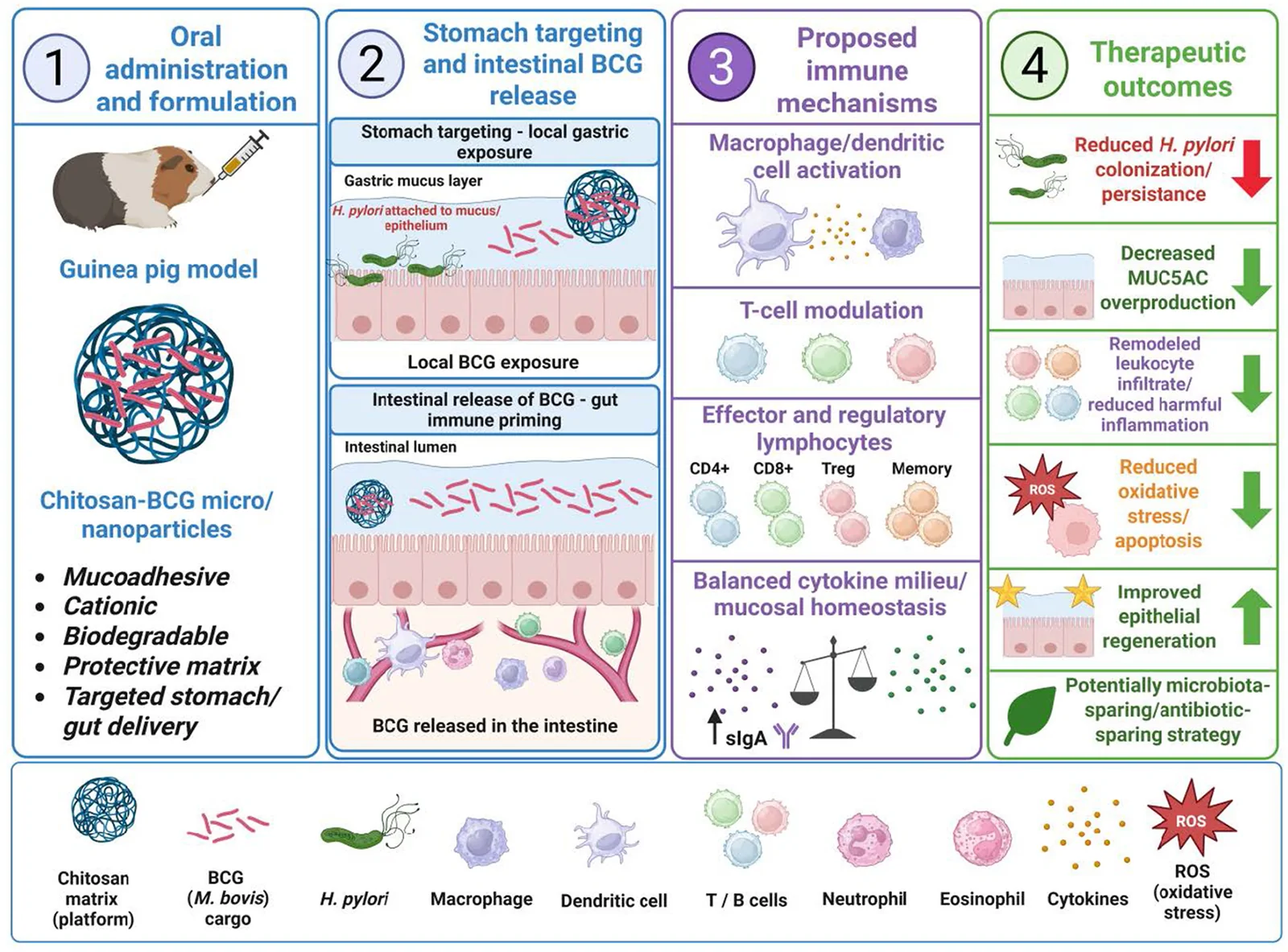
Proposed mechanisms of experimental chitosan-*M. bovis* BCG micro-/nano-formulations facilitating the control of *H. pylori* colonization in the *Cavia porcellus* model. Orally administered chitosan-based micro/nanoparticles loaded with *M. bovis* BCG may adhere to gastric mucus, protect the mycobacterial cargo, and enable local BCG exposure in the stomach and/or delayed BCG release in the gut. In the stomach, mycobacteria may target *H. pylori*-colonized mucus, whereas in the gut they may support immune priming and immune training. The suggested immunomodulatory mechanisms include activation of macrophages and dendritic cells, modulation of T-cell subsets, induction of effector and regulatory lymphocyte responses, elevation of secretory immunoglobulin A (sIgA), and restoration of the pro- and anti-inflammatory cytokine balance. These processes may contribute to reduced *H. pylori* colonization, control of MUC5AC production, remodeling of leukocyte infiltration, reduced oxidative stress and apoptosis, and improved epithelial regeneration. The scheme summarizes *in vitro* studies and experimental data obtained in the *Cavia porcellus* model. BCG, *Mycobacterium bovis Bacillus Calmette-Guerin*; MUC5AC, mucin 5AC; ROS, reactive oxygen species; Treg, regulatory T cells; sIgA, secretory immunoglobulin A; *H. pylori, Helicobacter pylori*. Created in BioRender. Mikolajczyk-Chmiela (2026) (https://BioRender.com/8snqrwo) is licensed under CC BY 4.0.

Further studies support the diversity of chitosan-based anti-*H. pylori* approaches. These include native chitosan NPs evaluated *in vitro* and *in vivo*, pH-responsive chitosan/heparin formulations designed specifically for stomach-delivery, fucose-functionalized chitosan/heparin NPs loaded with amoxicillin, unmodified chitosan MPs capable of binding *H. pylori* and reducing gastric infection in mice, mannose-modified chitosan/poly(malic acid) NPs with antibacterial and anti-biofilm activity against multidrug-resistant *H. pylori*, and chitosan-based nanocomposites combining plant-derived mucilage or selenium NPs (Luo et al., 2009; Lin et al., 2009, 2013; Henriques et al., 2020; Arif et al., 2022; Aborabu et al., 2024) (Figure 1 and Table 1).

**Table 1:** Concepts of chitosan-based strategies against *H. pylori*: mechanisms, advantages, and translational gaps.

Strategy	Active component/cargo	Main mechanism	Expected benefit	Key translational gap	References
Antibiotic-loaded chitosan formulations	Amoxicillin, clarithromycin, metronidazole	Mucoadhesion, protection of antibiotics at acidic pH, prolonged gastric retention and local drug release	Enhanced local antibiotic exposure and potentially improved eradication efficacy	Limited activity against resistant strains; need for *in vivo* pharmacokinetic and pharmacodynamic validation	Arora et al., 2011; Lin et al., 2009, 2013; Yao et al., 2022; Khoshnood et al., 2023
Chitosan with natural antimicrobial compounds	Curcumin, resveratrol, aloe vera, berberine, eugenol, *Acacia seyal* extract, broccoli mucilage/selenium nanocomposites and other phytochemicals	Enhanced solubility and stability of bioactive compounds with antibacterial, antioxidant and anti-inflammatory activity	Antibiotic-sparing or adjunctive therapy aimed at reducing bacterial load and mucosal inflammation	Variable composition, uncertain dosing, formulation stability and limited clinical evidence	Lin et al., 2015; Yahya et al., 2022; Hidalgo et al., 2023; Spósito et al., 2024; Aborabu et al., 2024; Tahmasebi et al., 2026; Al-Rajhi et al., 2026
Chitosan with antimicrobial peptides	CM11 and other antimicrobial peptides	Peptide stabilization, targeted local delivery and disruption of bacterial membranes	Alternative approach against resistant *H. pylori* strains	Peptide degradation, possible cytotoxicity, production cost and limited *in vivo* validation	Moosazadeh Moghaddam et al., 2023
Native and modified chitosan targeting urease, adhesion or bacterial biofilm	Chitosan alone or glycan/lectin-modified, fucose-modified, mannose-modified, carboxymethylated, thiolated or trimethylated chitosan formulations	Interference with bacterial urease activity, adhesion, membrane integrity and biofilm formation; improved mucoadhesion, solubility or prolonged gastric retention depending on chitosan derivatization	Anti-virulence approach targeting persistence rather than only bacterial viability	Need to connect anti-virulence effects with decreased colonization, inflammation and recurrence *in vivo*; derivative-dependent toxicity, reproducibility and scale-up require validation	Chang et al., 2020; Lai et al., 2022; Chitas et al., 2024; Zhang et al., 2025; Luo et al., 2009; Henriques et al., 2020; Arif et al., 2022
Chitosan-based mucosal vaccine formulations	*H. pylori* antigens, DNA vaccines or multi-epitope constructs	Protection of antigenic cargo, improved mucosal adsorption and stimulation of local immune responses; induction of mucosal IgA and systemic immune responses	Preventive or therapeutic immune strategy	*H. pylori* vaccine; unclear immune correlates of protection and durability	Renu and Renukaradhya, 2020; Gaglio et al., 2023; Amaral et al., 2025; Chan et al., 2025
*M. bovis* BCG-loaded chitosan micro/nanoparticles	Live or inactivated *M. bovis* BCG or *M. bovis* BCG-derived immunomodulatory components	Gastric and intestinal delivery of immunomodulatory cargo; activation of innate and adaptive immunity; modulation of macrophages, dendritic cells and T lymphocytes; induction of secretory IgA; reprogramming of the gastric immune environment	Host-directed, antibiotic-sparing approach; decreased experimental *H. pylori* colonization in the *Cavia porcellus* model; potential support of mucosal regeneration processes	Requires systematic validation of safety, dose, viability or antigenic load, live vs. inactivated BCG, delivery site, immune balance, microbiota effects, recurrence prevention and clinical translatability	Caetano et al., 2017; Netea et al., 2016; Chen et al., 2023; Gonciarz et al., 2023a,b, 2024a,b, 2026a,b

## 4 Loading of chitosan-derived carriers with antimicrobial or anti-inflammatory components

### 4.1 Encapsulation of antibiotics, natural compounds, and antimicrobial peptides

Chitosan carriers are frequently considered as systems that may enhance the local efficacy of antibiotics used in anti-*H. pylori* eradication therapy, including clarithromycin and metronidazole. Their advantages are related to chitosan mucoadhesion, which can maintain higher drug concentrations in the mucus, reduce rapid washout, and limit the need to increase systemic dosage (Chaves de Souza et al., 2020; Rehman et al., 2023).

Another therapeutic approach involves chitosan formulations containing plant-derived compounds, including curcumin, eugenol, essential oil components, polyphenols, or plant extracts. These polyphenol-enriched substances may exert antibacterial and anti-inflammatory effects and help neutralize oxidative stress (Hidalgo et al., 2023; Yahya et al., 2022).

Recent studies using experimental histological and molecular models have shown that eugenol-loaded chitosan NPs reduced *H. pylori*-driven abnormalities (Tahmasebi et al., 2026), whereas *Acacia seyal* seed extract attenuated *H. pylori*-induced oxidative stress. Molecular docking further suggested possible interactions between extract components, chitosan, and *H. pylori* urease (Al-Rajhi et al., 2026).

Antimicrobial peptides are short, usually cationic and amphipathic molecules produced by host cells or designed synthetically to damage bacterial membranes, interfere with intracellular targets, and modulate immune responses. Their relevance for anti-*H. pylori* therapy is related to mechanisms that differ from those of conventional antibiotics. Chitosan nanocarriers can protect antimicrobial peptides against enzymatic degradation, improve their retention at mucosal surfaces, and enhance local release in the stomach. For example, CM11-loaded concanavalin A-coated chitosan NPs have been proposed for targeted treatment of *H. pylori* SS1 infection *in vitro* and in a mouse gastric infection model (Moosazadeh Moghaddam et al., 2023). Nevertheless, peptide stability, possible cytotoxicity, production cost, and limited *in vivo* validation remain important barriers.

### 4.2 Neutralization of *H. pylori* virulence strategies

Approaches designed to weaken *H. pylori* mechanisms responsible for survival and colonization are increasingly considered potential anti-*H. pylori* strategies. Most studies still focus on classical antimicrobial outcomes, such as minimum inhibitory concentration, bacterial count reduction, urease activity, adhesion, and biofilm formation. Chitosan and chitosan-derived formulations have been evaluated *in vitro* using reference and clinical *H. pylori* strains, as well as an adhesion model based on human gastric carcinoma TSGH9201 cells (Chang et al., 2020). Other targeted nano- and microsystems affecting adhesion, UreI-dependent delivery, bacterial membranes, or biofilm-associated barriers have been reviewed by Chitas et al. (2024). By contrast, modulation of immune responses, resolution of inflammation, restoration of epithelial barrier integrity, and mucosal regeneration have been assessed less frequently (Khoshnood et al., 2023).

A recent study by Chan et al. demonstrated the development of a chitosan NP-based oral DNA vaccine encoding the *H. pylori* urease B subunit. The formulation was stable under simulated gastric and duodenal conditions, induced mucosal IgA and systemic IgG responses in a mouse model, increased interferon-gamma and interleukin-17 production, and reduced colonization (Chan et al., 2025). The usefulness of chitosan-based formulations is not limited to *H. pylori*; such systems have also been assessed in respiratory, intestinal, and wound-related infection models (Renu and Renukaradhya, 2020; Gaglio et al., 2023; Aibani et al., 2021). However, mechanisms and biosafety profiles are infection- and tissue-specific; therefore, data from other models should be treated as supportive but cannot be directly generalized to *H. pylori* therapy.

## 5 *M. bovis* BCG-loaded chitosan formulations: potential immunomodulatory effects against *H. pylori*

### 5.1 Why *M. bovis* BCG?

The use of chitosan formulations containing live *M. bovis* BCG bacilli can be considered an immunomodulatory strategy to support the control of *H. pylori* colonization, infection-induced inflammation, and bacterial immune evasion. This concept is based on both the biological properties of *M. bovis* BCG and the characteristics of chitosan as a mucoadhesive carrier (Netea et al., 2020; Chen et al., 2023; Gonciarz et al., 2023a,b, 2024a).

*Mycobacterium bovis* BCG is a live attenuated vaccine strain that induces protection against tuberculosis. BCG mycobacteria may participate in trained immunity, defined as functional reprogramming of innate immune cells, particularly monocytes and macrophages, through epigenetic and metabolic changes (Netea et al., 2020; Chen et al., 2023). As a result, reprogrammed innate immune cells, together with adaptive immune responses, may respond more effectively to subsequent exposure to homologous or unrelated pathogens, including *Staphylococcus aureus, Streptococcus spp., Candida albicans*, rhinoviruses, and coronaviruses (Kleinnijenhuis et al., 2012; Kang et al., 2023; Arts et al., 2018; Moorlag et al., 2022). Early BCG vaccination of newborns has also been associated with long-lasting innate immune memory in school-aged children (Jurczak et al., 2025). Based on its immunomodulatory activity, BCG has also been used as an adjuvant therapy for bladder cancer (Morales et al., 1976; Yamazaki-Nakashimada et al., 2020).

Oral administration of live mycobacteria requires a carrier that can support bacterial survival, persistence, and activity in the acidic gastric milieu. As a mucoadhesive material, chitosan interacts with negatively charged mucus and can therefore prolong formulation retention in the stomach, limit rapid removal from the gastrointestinal tract, and improve local mucosal exposure to *M. bovis* BCG. Gonciarz et al. (2024a) developed biocompatible spray-dried, pH-sensitive chitosan MPs containing *M. bovis* BCG and modified with N-acetyl-D-glucosamine or Pluronic F127 to release live mycobacteria in the stomach and gut, thereby facilitating mucosal immune stimulation.

In *Cavia porcellus* orally inoculated with chitosan MPs loaded with *M. bovis* BCG, mucosal *H. pylori* colonization was reduced, as shown by quantitative polymerase chain reaction targeting the cagA gene. This effect was accompanied by reduced MUC5AC deposition, greater macrophage infiltration, increased macrophage phagocytic activity, and elevated secretory IgA in gastric tissue (Gonciarz et al., 2023a,b, 2026a). Activation of splenic germinal centers further supported the induction of an adaptive humoral response (Gonciarz et al., 2026a). Exposure to chitosan MPs loaded with *M. bovis* BCG also increased the gastric deposition of effector CD4+ and CD8+ T lymphocytes, regulatory Foxp3+ lymphocytes, and eosinophils (Gonciarz et al., 2026b). These cell populations may reflect a controlled effector-regulatory response that could limit harmful inflammation, reduce bacterial adhesion, and support epithelial repair. However, the rationale for using *M. bovis* BCG-loaded chitosan formulations should be interpreted as an experimental host-directed and immunomodulatory concept rather than as a proven clinical anti-*H. pylori* therapy.

Importantly, the clinical efficacy of chitosan-based formulations, including BCG-loaded chitosan MPs and NPs, against *H. pylori* infection in humans has not yet been demonstrated. Therefore, these formulations should currently be considered experimental and potentially adjunctive rather than clinically validated therapeutic solutions.

Despite promising data, chitosan formulations containing *M. bovis* BCG have not yet been systematically studied as a strategy against *H. pylori*. Important unresolved issues include comparisons among different chitosan modifications, *M. bovis* BCG doses, local and systemic release kinetics, administration timing, and long-term effects on mucosal immune priming, including trained immunity, T- and B-lymphocyte responses, eosinophil infiltration, tissue regeneration, and microbiota composition. Future studies should therefore combine traditional endpoints, such as bacterial load and inflammatory activity, with analyses of trained immunity, effector and regulatory T-cell activity, epithelial barrier integrity, and tissue repair markers. Such immune activation may be beneficial only if it does not aggravate chronic gastritis.

### 5.2 Translational challenges

Translating chitosan formulations containing *M. bovis* BCG into clinical applications requires addressing several issues related to biocompatibility, standardization, and control of immune activation. It is worth noting that the oral route of BCG delivery was historically used in humans vaccinated against tuberculosis in 1921 (Starshinova et al., 2025). However, *M. bovis* BCG formulations intended for oral administration to control *H. pylori* infection in humans require separate assessment of local and systemic biosafety, particularly in relation to dissemination of vaccine mycobacteria, excessive inflammation, and possible risks in immunocompromised individuals (World Health Organization, 2018, 2024). The biological form of *M. bovis* BCG is also important. Live mycobacteria may induce stronger trained immunity and immune responsiveness, but they carry greater biological risk. Inactivated mycobacteria, bacterial lysates, cell wall fractions, or selected antigenic components may offer safer alternatives while retaining some immunomodulatory properties (Chen et al., 2023). However, each approach requires different standardization: for live *M. bovis* BCG, viability and colony-forming units must be controlled, whereas inactivated *M. bovis* BCG or mycobacterial fractions require quantitative characterization of antigenic load and reproducibility of immune activation.

Despite the growing number of studies on chitosan-based anti-*H. pylori* formulations the available evidence remains fragmented and is derived mainly from *in vitro* experiments and selected animal models. Moreover, some specialized concepts, as BCG-loaded chitosan formulations, have been investigated by only a limited number of researchers. Therefore, these approaches should currently be regarded as experimental and preclinical and require broader independent validation before clinical translation.

## 6 Future perspective and conclusions

Future treatment strategies for *H. pylori* infection should gradually shift from traditional eradication regimens based mainly on antibiotics and proton pump inhibitors toward approaches that simultaneously target the pathogen, gastric mucosa, inflammatory milieu, and host immune system. In the context of increasing resistance of *H. pylori* isolates to commonly used antibiotics, such approaches may help reduce recurrence and support recovery of mucosal function after treatment (Malfertheiner et al., 2022; Albush et al., 2025).

Chitosan may play a versatile role in this context. First, it enables local delivery of antimicrobial components, including antibiotics, natural compounds, and antimicrobial peptides, thereby increasing their retention in the stomach and contact with the mucosal layer (Chaves de Souza et al., 2020; Khoshnood et al., 2023; Moosazadeh Moghaddam et al., 2023). Second, because of its mucoadhesive properties and positive surface charge, chitosan may support the neutralization of selected *H. pylori* virulence mechanisms, including urease activity, adhesion, and biofilm formation (Chang et al., 2020; Chitas et al., 2024). Chitosan carriers loaded with plant extracts enriched in polyphenols, either alone or in combination with antibiotics or other therapeutics, may help control *H. pylori* and attenuate the inflammatory response (Yahya et al., 2022).

Chitosan formulations containing *M. bovis* BCG are a promising preclinical concept because they combine mucoadhesive carrier properties with the ability of mycobacteria to reprogram mucosal immunity, activate innate and adaptive immune responses, and potentially support gastric tissue regeneration (Gonciarz et al., 2024a, 2026b). These approaches are intended to reduce bacterial load, rebuild the epithelial barrier, limit inflammation, and restore mucosal immune balance. An additional potential benefit of locally targeted strategies is reduced systemic antibiotic exposure and better preservation of the gut microbiota. Chitosan-derived formulations loaded with natural compounds, antimicrobial peptides, or immunomodulatory *M. bovis* BCG may therefore represent adjunctive approaches to limit antibiotic use in the management of *H. pylori* infection, because they target both the pathogen and the mucosal milieu.

Recent data on chitosan-based oral DNA vaccines, carboxymethyl chitosan gastroretentive formulations, and chitosan NPs loaded with eugenol or plant extracts indicate further progress from simple antibiotic delivery toward multifunctional gastric formulations combining antimicrobial, anti-virulence, immunomodulatory, and tissue-protective effects (Chan et al., 2025; Zhang et al., 2025; Tahmasebi et al., 2026; Al-Rajhi et al., 2026).
